# Second-Generation 3D Automated Breast Ultrasonography (Prone ABUS) for Dense Breast Cancer Screening Integrated to Mammography: Effectiveness, Performance and Detection Rates

**DOI:** 10.3390/jpm11090875

**Published:** 2021-08-31

**Authors:** Gianluca Gatta, Salvatore Cappabianca, Daniele La Forgia, Raffaella Massafra, Annarita Fanizzi, Vincenzo Cuccurullo, Luca Brunese, Alberto Tagliafico, Roberto Grassi

**Affiliations:** 1 Dipartimento di Medicina di Precisione Università della Campania “Luigi Vanvitelli”, 80131 Napoli, Italy; gianluca.gatta@unicampania.it (G.G.); salvatore.cappabianca@unicampania.it (S.C.); vincenzo.cuccurullo@unicampania.it (V.C.); roberto.grassi@unicampania.it (R.G.); 2 IRCCS Istituto Tumori “Giovanni Paolo II”, 70124 Bari, Italy; r.massafra@oncologico.bari.it (R.M.); a.fanizzi@oncologico.bari.it (A.F.); 3 Dipartimento di Medicina e Scienze della Salute “Vincenzo Tiberio”—Università degli Studi del Molise, 86100 Campobasso, Italy; luca.brunese@unimol.it; 4 Department of Health Sciences, University of Genoa, 16126 Genoa, Italy; alberto.tagliafico@unige.it

**Keywords:** 3D prone ABUS, breast density, breast cancer detection, breast cancer screening

## Abstract

In our study, we added a three-dimensional automated breast ultrasound (3D ABUS) to mammography to evaluate the performance and cancer detection rate of mammography alone or with the addition of 3D prone ABUS in women with dense breasts. Our prospective observational study was based on the screening of 1165 asymptomatic women with dense breasts who selected independent of risk factors. The results evaluated include the cancers detected between June 2017 and February 2019, and all surveys were subjected to a double reading. Mammography detected four cancers, while mammography combined with a prone Sofia system (3D ABUS) doubled the detection rate, with eight instances of cancer being found. The diagnostic yield difference was 3.4 per 1000. Mammography alone was subjected to a recall rate of 14.5 for 1000 women, while mammography combined with 3D prone ABUS resulted in a recall rate of 26.6 per 1000 women. We also observed an additional 12.1 recalls per 1000 women screened. Integrating full-field digital mammography (FFDM) with 3D prone ABUS in women with high breast density increases and improves breast cancer detection rates in a significant manner, including small and invasive cancers, and it has a tolerable impact on recall rate. Moreover, 3D prone ABUS performance results are comparable with the performance results of the supine 3D ABUS system.

## 1. Introduction

Numerous studies have proven that, in the last 30 years, cancer mortality has decreased; this is predominantly due to an increase in mammography screening programs. Given the diminishing number of late-stage cancers, it should be clear that mortality has also diminished, with statistics reporting a 45% decrease in death rates [1]. Progress in recognizing genetic markers and risk causes for cancer notwithstanding, about 70/80% of women are still affected without showing any considerable predictive factors [2,3]. The main plan of action for decreasing and reducing cancer mortality by increasing early detection remains the screening program.

Standard mammography breast cancer screening has helped to reduce breast cancer mortality [4]. That said, the number of cancers not detected during a mammogram is substantial, especially when it comes to women with dense breasts, with a sensitivity value of 30–48% [5]. Mammography density refers to the percentage of dense tissue in an entire breast and is based on the appearance of digital mammography based on different attenuation characteristics of X-rays as a function of the composition of the breast tissue (radiolucent and dark fat tissue, glandular tissue and connective tissue radiopaque and white) [6]. In a dense mammography, therefore, glandular and connective tissue will prevail significantly in terms of percentages compared to adipose tissue. The most common system to classify the composition of a breast, and therefore its density, is currently BI-RADS of the American College of Radiology (ACR) [7]. A breast’s density has proven to be a determinant breast cancer factor [8]; dense breasts are also quite frequently found in women. Roughly 2/3 of premenopausal women and about 30% of older women have a higher breast density of around 50%. It has been noted that the number of cancers and cancer intervals not detected by mammography are much higher for women with dense breasts rather than fat breasts. Using digital mammography rather than analogic mammography shows a 55 to 70% increase in cancer detection rates [9]. However, a large number of breast cancers still remain undetected. Even when adding tomosynthesis to digital mammography, detection rates of dense breast cancer are lower than the detection rates of ultrasound alone [10]. For a long time, the manual, handheld ultrasound (HHUS) has proven itself to be a valuable and effective add-on diagnostic screening tool, which allows for the detecting of cancer in women with dense breasts. This technique is not hindered by the density of breast tissue [11]. Some studies have obtained a significantly higher detection rate in automated breast ultrasounds (3D ABUS) compared to HHUS [12]. SomoInsight is the most extensive study that has assessed the diagnostic performance of 3D ABUS in a screening scenario, including in 15,318 asymptomatic women with dense breasts. By associating 3D ABUS with full-field digital mammography (FFDM), the increase in detection rate was 1.9 per 1000 women, thus increasing sensitivity. Moreover, 3D ABUS associated with FFDM plays an important role in screening programs [12]. Berg et al. [13] have shown that the HHUS’s cancer detection rate is similar to that of FFDM, where more ductal carcinomas in situ (DCIS) were found with the FFDM, while more invasive node negative cancers were found using HHUS. Since 2007, magnetic resonance imaging (MRI) has been recommended by the American Cancer Society as a diagnostic screening technique to be used for women at a high risk (lifetime risk greater than 20–25%) of breast cancer [14], while HHUS still remains disputed and dubious as a screening tool.

Some studies have reported a good correlation between 3D ABUS and MRI for lesion measurement, while others have evaluated the performances of 3D ABUS second look and HHUS second look in detecting additional suspicious lesions in patients undergoing preoperative breast MRI. Second look 3D ABUS detected more injuries than HHUS, while 10% of injuries detected by 3D ABUS were not evident in HHUS [12].

Plentiful studies [14,15,16,17,18,19] have already determined, verified, and validated the supine 3D ABUS. It differs from the HHUS for following up a standardized protocol that can be operated by medical and nonmedical personnel who have undergone a brief training period. Moreover, 3D ABUS protocol does not require a highly experienced radiologist, acquiring 3D volumes that overlap, and it is therefore read and assessed in multiplanar sections. Images are acquired in a transverse plain with a 6–14 MHz linear array transducer; these images are then elaborated at the workstation, where the sagittal and coronal plain can be reconstructed.

The aim of our work is to compare the performance and detection rate of mammography alone versus double mammography—3D ABUS detection. Regarding the fact that the second-generation 3D automatic breast ultrasound system involved patients being in a prone rather than a supine position, our studio appears to be the first study to carry out this type of assessment.

## 2. Materials and Methods

This study has been evaluated and approved by the Regional Ethics Review Board (registration number 189.20). This study is carried out in the manner prescribed by the Helsinki Statement. Study-specific informed consent was signed by each patient enrolled.

### 2.1. Study Population

All patients involved have signed their consent to use the data. Patients’ signed consent clearly states that the 3D ABUS was not a mammography substitute but an additional investigational analysis procedure. From June 2017 to February 2019, all women who came for routine mammograms underwent a selection process to be enrolled in our study. Follow-up of all enrolled patients covered a period of 24 months (February 2021). We chose only those women who fell into the following categories: women with breast imaging reporting and data system (BI-RADS) density 3/4; heterogeneously or extremely dense breasts; with no implants; not pregnant or breast feeding; with neither personal nor familial cancer history; within the age bracket of 40 to 75; not showing clear breast cancer symptoms; and those who had undergone surgery and percutaneous biopsy in the previous year (Table 1).

The density BI-RADS category was assessed during the screenings by radiographers who received specific training, or by the first radiologist reader if present at the screening. Based upon the above selection criteria, the eligible participants included in our study totaled 1165.

### 2.2. FFDM

The equipment used on all the patients who underwent mammography was FFDM Mammomat Inspiration (Siemens Erlangen Germany). The first mammography examination covered 3 different projections (six images): craniocaudal (CC), mediolateral oblique (MLO) and mediolateral (ML) views, while subsequent examinations were performed with only two images (MLO and CC).

### 2.3. 3D Prone ABUS

The automated breast ultrasound system equipment used was the prone system “Arietta/Sofia” (Hitachi Tokyo, Japan). Two out of the three radiologist who took part in the trial were specifically trained by 3D ABUS professional technicians in order to be fully qualified to best utilize the system’s capacities, while the third radiologist was trained by the other two radiologists. Sofia is a 3D scan system device made of three different parts: a scan bed, a probe and a workstation. The scan bed table (on which the patient lies) has a 184 mm circular area in which a 92 mm long multifrequency (5–10 MHz) ultrasound transducer is embedded. The probe is sealed underneath a glass layer on which the ultrasound gel lotion is applied. A specific and dedicated workstation elaborates all of the scanned images as 3D images. The pre scan touch panel interface option allows the parameter to be modified (gain, depth, focus, etc.)

**Figure 1:**
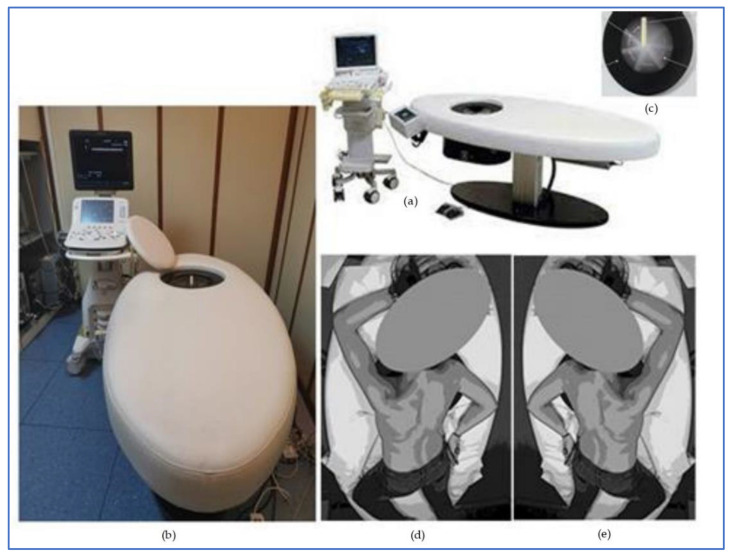
3D Prone ABUS device and patient optimized positioning. (**a**) 3D Prone ABUS graphic design drawing. (**b**) 3D Prone ABUS hospital device picture. (**c**) Table embedded transducer. (**d**) Right breast optimized patient scanning position. (**e**) Left breast optimized patient scanning position.

A patient lies down prone with the bent contralateral leg positioned slightly rotated so that the breast flattens on the glass, with the nipple centered in the cone; the arm can be placed on the side (Figure 1). The operator can easily ascertain whether the patient is in the right position in order for the examination to start. Once the patient is well positioned and still, the transducer automatically rotates around the breast in a clockwise circular motion to capture a 360° breast volume scan image in one attempt (approximately 35 s per breast). The probe automatically goes back to its home position when the pre-scan is complete. Correct positioning of the patient is vital, as their incorrect placement might alter the examination’s results; for instance, if the entire breast is not fully covering the scanning area, an image drop-off might occur. The operator can apply gentle pressure to the patient’s back in order to avoid any lack of contact between the glass and the breast, as this might generate artefacts.

The original acquisitions were achieved in the transverse plane—the same as in supine ABUS systems—and they are then rebuilt in the sagittal and coronal planes.

The process takes roughly 10 min per patient before the data are sent to the workstation. Three experienced breast radiologists (10 to 20 years’ experience) read and interpret the images; two of the three radiologists were fully trained by a Sofia ABUS technician, while the third one was trained by the other two radiologists.

### 2.4. Interpretation of Images

For each case, two of the three radiologists involved in the study sequentially interpreted both the mammogram and the 3D prone ABUS. In case of a reading of concern, none of the radiologists specified whether the concern was about full-field digital screening mammography (FFDSM) or 3D ABUS, nor did they discuss the position of the finding. This was executed to perform a double-blinded reading and to enable the double evaluation of BI-RADS results of the 3D ABUS prone exam and establish inter-observer variability.

To evaluate possible malignant cases or possible recalls, each radiologist evaluated the FFDSM exams on the same workstation (5 Mega Pixel resolution), without access to the 3D prone ABUS images. Only after assigning the final Bi-RADS opinion to the screening mammography did the same radiologist interpret the 3D prone ABUS images and express a final opinion based on the assessment of mammography + 3D prone ABUS. All three radiologists evaluated 3D ABUS images on the same reconstruction workstation. Therefore, for each case, the mammography images and the three-dimensional ABUS images were evaluated in a double reading by two radiologists, with a determination for the final consensus.

The radiologists interpreted the 3D ABUS findings (and the FFDSM images when recall cases occurred) with respect to the following parameters: shape of mass, margins, orientation, echo pattern, lesion boundary and posterior acoustic features. Moreover, 3D prone ABUS System BI-RADS classification was assessed with the following criteria: 1 = negative; 2 = benign; 3 = probably benign; 4a = low suspicion; 4b = moderate suspicion; 4c = high suspicion; 5 = suggestive of malignancy. Readings of the BI-RADS images’ concordance were assigned by the K Fleiss statistic.

We recalled all women with suspicious findings detected in either the 3D ABUS or FFDSM to perform detailed mammography and/or handheld ultrasonography exams. After the recall, all women for whom no signs of malignancy were found were informed, and they were asked to come back for their next screening following normal timings and procedures (dense breast annual checkup). In case of suspected malignancy, women were subjected to biopsy; the removed tissue was then analyzed by a pathologist to provide a final and confirmed diagnosis. For examinations evaluated with BI-RADS 1 or 3, an additional imaging investigation was carried out in order to determine a final and defined BI-RADS assessment. When the assigned BI-RADS was 4 or 5, a stereotactic or percutaneous guided ultrasound biopsy was performed. In case of benign biopsy results, no further investigations were required. If a malignancy was proven by biopsy results, the lesions were surgically removed.

In each observation group (mammography versus mammography + 3D ABUS), deviations in cancer detection rate, recall rate, sensitivity, specificity, positive predictive value (PPV) and negative predicative value (NPV) were evaluated. We assigned the women into three groups: PPV1 (proportion of tumors detected in women recalled for further investigation rather than routine screening), PPV2 (proportion of cancer detection among women recommended for biopsy), and PPV3 (proportion of cancer detection among women undergoing biopsy). All recommended biopsies were performed, and PPV2 and PPV3 were matched.

McNemar’s test was utilized to compare diagnostic yield between mammography and automated Sofia ultrasound system. Results below 0.05 were classified as statically significant. Analyses were carried out using IBM Spss Statistics 23 software [20].

### 2.5. Statistical Analysis

Categorical and continuous variables were assessed using a Clopper–Pearson chi-square test and a Student’s *t*-test. Sensitivity and specificity calculations, against both mammography and the 3D prone ABUS system, were determined using a 2 × 2 table. McNemar’s SPSS was used for specificity testing and binomial testing was used to assess sensitivity. To evaluate PPV, we took into account only the recalled patients, and three different American College of Radiology (ACR) classification values were applied as follows: (a) Cancer percentage found at screening but where further investigation was needed (PPV1); (b) cancer percentage where biopsy was requested (PPV2); and (c) proportion of detected cancer after biopsy (PPV3). All requested biopsies were performed; therefore, the estimated PPV2 and PPV3 results were identical. No patient skipped the follow-up. Our study statistics results were compared to the BI-RADS classification using the Fleiss statistic K test with the Landis and Koch scale value. Diagnostic estimates interval of confidence applied was 95%; SPSS (IBM CORPORATION SPSS Statistics 23) was used for our analysis.

## 3. Results

The selected patients divided by age group and mammographic density are shown in Table 1.

**Figure 2:**
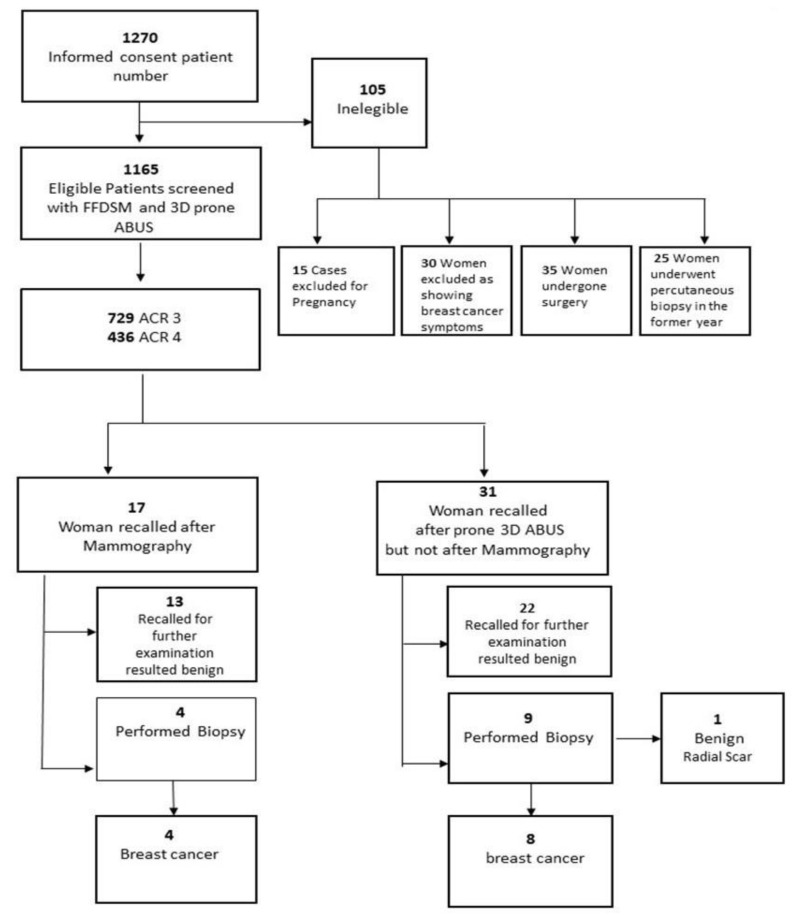
Study overview flowchart.

Among the patients selected, twelve women were diagnosed with breast cancer, including four cancers detected with mammography alone, while another eight cancers were detected by adding 3D prone ABUS (Figure 2, Table 2). In total, 1270 asymptomatic women were enrolled in the study but 105 were excluded (8.3% of 1270); among the latter, 15 patients (15%) were pregnant, 30 patients (29%) had clear symptoms of breast cancer, 35 patients (34%) had recently undergone breast surgery and 25 patients (24%) had undergone biopsy within the same year. Finally, 1165 women were selected as suitable; 729 patients (62%) were classified as ACR3 and 436 (37%) were classified as ACR4. The average age of the ACR3 group was 47, while that of the ACR3 group was 50. Most of the other clinical and demographic characteristics among the two groups (ACR3 and ACR4) were similar.

### 3.1. Diagnostic Accuracy

By adding 3D prone ABUS to mammography, screening sensitivity saw of a 34.7% increase (95% CI: 16.3–61.2; *p* ˂ 0.001). However, the addition of interval cancer resulted in a sensitivity increase of 31.8% (95% CI: 11.7–54.6; *p* ˂ 0.001).

When performing mammography in conjunction with 3D prone ABUS, biopsyscreening rates showed a 7% increase in 1000 women (95% confidence interval CI:4.3–8.8; *p* ˂ 0.001), while surgery increased by a rate of 3.4% (95% CI: 1.6–6.1; *p* ˂ 0.007). There were fewer biopsies (PPV2 and PPV3) requested and carried out after performing mammography in conjunction with 3D prone ABUS than there were compared to those requested and carried out after performing mammography alone: 41% (95% CI:26.4–59.2; *p* ˂ 0.001) in relation to the two examinations versus 58.8% (95% CI:30.9–78.3; *p* ˂ 0.001) for mammography. Furthermore, the inclusion of interval cancer in the NPV increased in mammography combined with 3D prone ABUS in comparison to mammography alone, though only by 0.2% (95% CI:0.12–3.1) (Table 3).

### 3.2. Cancer Detection

**Figure 3:**
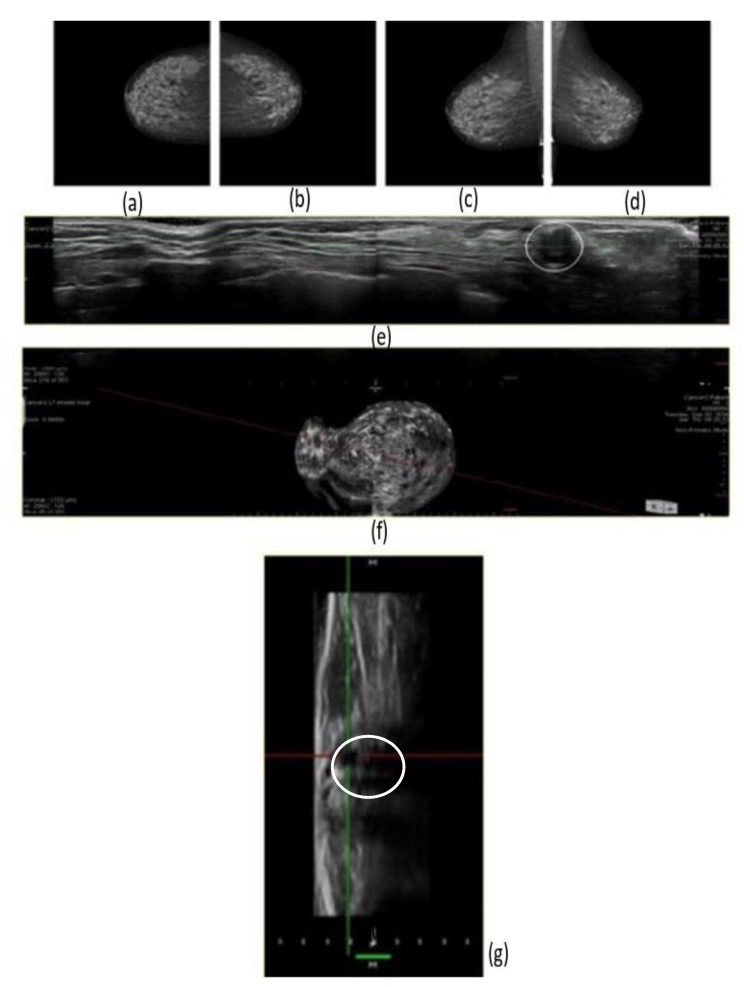
3D Prone ABUS Images of ductal invasive cancer which was not visible on dense breast mammography. (**a**) Full field digital right craniocaudal breast mammography. (**b**) Full field digital left craniocaudal breast mammography. (**c**) Full field digital right mediolateral-oblique breast mammography. (**d**) Full field digital left mediolateral-oblique breast mammography. (**e**) 3D Prone ABUS right breast transversal plane image showing node negative invasive ductal cancer (white circle). (**f**) 3D Prone ABUS right coronal plane reconstruction. (**g**) 3D Prone ABUS Sagittal plane reconstruction cancer image circumscribed by perpendicular plane axis.

**Figure 4:**
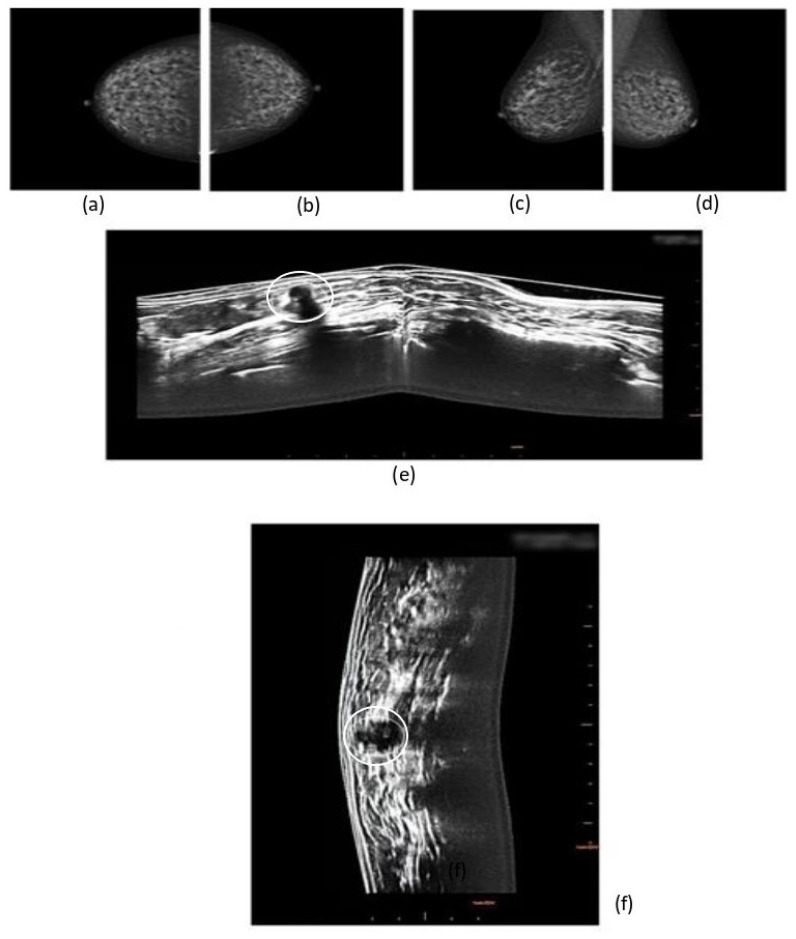
3D Prone ABUS Images of ductal invasive cancer not detect with the mammography. (**a**) Full field digital right craniocaudal breast mammography. (**b**) Full field digital left craniocaudal breast mammography. (**c**) Full field digital right mediolateral-oblique breast mammography. (**d**) Full field digital left mediolateral-oblique breast mammography. (**e**) 3D Prone ABUS right breast transversal plane image showing node negative invasive ductal cancer (white circle). (**f**) 3D Prone ABUS left sagittal plane reconstruction (white circle).

In our study. eight cancers were diagnosed with 3D prone ABUS (95% CI: 3.45–15.76; *p* ˂ 0.001). while four cancers were detected with the mammographic screening alone (95% CI: 1.09–10.24; *p* ˂ 0.001) (Table 4). The difference in results highlights the fact that. out of 1000 women. 4 more additional cancer were found (95% CI: 1.09–10.24; *p* ˂ 0.001). Consequently. there was an increase of 50% in terms of cancer detection (Figure 3 and Figure 4).

### 3.3. Recall Rate and Interval Cancers

In our study, the recall rate of mammography alone is 14.5 per 1000 women (95% CI: 9.0–19.8; *p* ˂ 0.001) and 26.6 per 1000 women for mammography combined with 3D prone ABUS (95% CI: 16.2–30.0; *p* ˂ 0.001); this is a difference in recall of 12.1‰ (95% CI: 4.0–39.9; *p* ˂ 0.001). During mammography screening, four interval cancers (0.3%) were (95% CI: 1.09–10.24; *p* ˂ 0.001), and they were only recognized only after patients perceived some irregularity during self-palpation. Three of four interval cancers were found in patients classified as ACR, while the fourth one was ACR3. In retrospect, we hypothesize that two interval cancers (ACR4) might have been spotted due to an unstructured opacity (obscured view in part of the breast) that was placed where the cancer developed, but it showed a low cancer predictive value.

### 3.4. BI-RADS Analysis

The BI-RADS analysis correlation of the three readers was perform, on 112 3D prone ABUS records (Table 5). All of the readers found eight cancers. As such. K Fleiss statistical analysis was applied to evaluate the data, which highlighted a substantial agreement between the three radiologists (0.72).

## 4. Discussion

Breast cancer is the most common cancer in women and the most common cause of cancer death worldwide wide, with an estimated 2.1 million cases diagnosed and more than 620.000 global deaths in 2018 [21]. There is therefore an increasing requirement to improve and extend breast-screening programs that have been shown to reduce breast cancer mortality by up to 45% [22]; however, screening mammography sensitivity is limited by dense breast, for which it is reduced by up to 48% in patients with extremely dense breasts (ACR category D) [23]. In addition, a recent publication [24] sheds new light on the density–breast biomarker by observing that patients with premenopausal fat breast (ACR category A) show significantly more adverse cardiovascular events (MACE) during the 10 years of follow-up. On the contrary, it is widely reported in the literature that mammographic density alone is an independent risk factor for breast cancer. with a relative risk in the course of life that can be estimated as being up to six times higher than in non-dense breasts [17]. This is the context in which the industry operates by proposing a new generation of ultrasound systems with prone patient positioning. The main purpose of our study was to assess the impact, results, related benefits or possible disadvantages regarding the cancer detection rate for women with dense breasts obtained by adding the 3D prone ABUS to mammography. We did not intend to measure or compare one examination against the other but, instead, to examine the advantage of combining them. thus highlighting their differences and complementary results. When it comes to women with dense breasts who are at a high risk of developing breast cancer [25]. 3D prone ABUS is undoubtedly more adept in terms of cancer detection compared with mammography alone [8,26].

Our study shows a high sensitivity of the prone ABUS system associated with mammography (93.5%). which is comparable to the sensitivity found in the more famous Somoinsight study (ABUS supine. 100%) [17]. This finding indicates that both positions (supine and prone) are reliable in terms of increasing mammographic sensitivity. The Somoinsight study, a multicenter prospective trial including 15.318 asymptomatic women with dense breast, compared mammography versus mammography plus 3D ABUS. A combined imaging approach led to an increase in sensitivity of 26.7%. Cancers detected only with 3D ABUS were significantly more likely to be invasive in comparison to those detected by screening with mammography alone (93.3 versus 62.2%. *p* = 0.001). Moreover. ABUS alone detected cancers that presented with a lower stage at diagnosis, suggesting positive prognostic implications. In this study, recall rates increased from 150.2 per 1000 women with mammography alone to 284.9 per 1000 women by adding ABUS, and specificity decreased by 13.4% for combined modalities (85.4% for mammography alone versus 72% for mammography plus 3D ABUS) [17,27]. Furthermore, in our study, we highlighted a significant increase in recall rate and biopsy by associating 3D ABUS with mammography alone; however, this was subsequently reduced with the progressive expertise of radiologists in relation to the new method, though this approach did remain superior to mammography alone.

In other studies in the literature [28,29,30], there were no significant variations in the detection of lesions between HHUS and ABUS, even if some showed a greater propensity of 3D ABUS to identify malignant lesions, depending on the size of and variations in the surrounding tissue; there was also a correlation in the results of the BI-RADS categories.

Our study shows a specificity of 87% with the addition of 3D ABUS, though this is reduced compared to mammography alone and is in line with other studies in the literature: it should be noted, however, that Choi [26] showed a higher diagnostic accuracy and specificity of 3D ABUS compared to HHUS (97.7 versus 96.5% diagnostic accuracy and 97.8 versus 96.7% specificity, respectively).

Moreover, an increase in cancer detection rates after the addition of prone ABUS to mammography (26.6 per 1000 women) is perfectly comparable with the results of other contributions in the literature that use the 3D ABUS supine system, though the number of patients enrolled should be taken into account [15,16,17]. On the other hand, we observed a 7% decrease in specificity with the addition of 3D ABUS compared to the specificity of mammography alone; this decrease in specificity is in line with the data presented in other published studies [16,17,27,31].Consequently, by using 3D prone ABUS, cancer detection rates increased within an acceptable recall rate of 26.6‰ against the 14.5‰ for mammography alone. The higher recall rate might be interpreted negatively, but we also need to consider the higher number of cancers detected as a justification for recall rates; as stated above, the recall rate is within an acceptable range. The detection rate improvement has been recognized also concerning dense breast women aged under 50 [32,33]. These data are in line and comparable with previous studies [15,34]. Moreover, 3D prone ABUS has some advantage from the perspective of radiologists, as the images have a higher definition, improved contrast and are sharper; they are also smaller, therefore reducing the elaboration delay at the computer monitor read station. Furthermore, we observed an important result regarding the radiologist’s K test BI-RADS evaluation accordance, which was (amongst 112 3D prone ABUS records) highly effective (K Fleiss test = 0.72). In terms of detection rate, by adding 3D prone ABUS to mammography for women with dense breasts, our study showed an increase of 3.4‰ in the cancers detected compared to mammography alone. These results are in line with and comparable to previous studies [13,35]. Although we compared our results to previous studies, we need to highlight the fact that, in our case, a patient’s prone position during the examination differentiates our results. Moreover, the examination device is different, as is the applied positioning technique. By implication, the authors hypothesize a benefit for the radiologist assessing the post MRI echography second look in relation to the images taken in the prone position during the MRI; however, these data fall outside the scope of this study and need to be confirmed by comparative studies. As also reported by other studies [17,36], we observed and experienced an increase in what concerns sensitivity when adding 3D prone ABUS to mammography, as sensitivity increased by 34.7%, which was lowered to 31.8% after taking into account interval cancers. The combining of mammography with 3D prone ABUS provides a sensitivity comparable to MRI but at a lower cost [37,38,39,40,41]. It is well known that MRI has been recommended by the American Cancer Society (ACS) for the screening of women at high risk of breast cancer. Although highly sensitive, it is an expensive diagnostic examination and may involve risks arising from the use of the contrast agent used; this agent is often poorly tolerated in patients undergoing examination, and some of them even have allergic reactions. In addition. MRI is characterized by a lower specificity than mammography, and often has a higher false positive rate; this leads to increased costs, as further diagnostic tests are required (e.g. biopsy) [18].

Possible future evolutions of the present study could concern comparisons between prone 3D ABUS and prone breast MRI, as well as comparisons between supine ABUS and supine breast MRI. As far as we know, there are no comparative studies of this type in the literature when analyzing large patient samples; a few of the existing studies focus on small samples of patients evaluated with supine ABUS and prone MRI for the assessment of density or in the search for residual disease in patients treated with neoadjuvant chemotherapy [42].

As far as we know, there are no comparative studies between 3D ABUS and MRI in the supine position, and there are only studies that take into consideration single methods of analysis [43,44].

Another possible development of this study could be a comparison of the performance of CAD systems associated with mammography [45,46,47,48,49] and that obtained by the association between mammography and 3D ABUS.

It is also possible to carry out comparative evaluations between ABUS and MRI with associated computed aided detection (CAD) in the latter method that partially reduce the visual disturbance effect on reporting caused by background parenchymal enhancement [50,51].

Another technique with the potential to become a valuable complement to mammography screening is tomosynthesis (quasi three-dimensional mammography).

In this regard, however, it is important to consider that multi-centric prospective trials ASTOUND and ASTOUND2 by Tagliafico et al.which compare the addition of HHUS and tomosynthesis in patients with dense breasts and negative mammography at screening, report a better diagnostic increase for echography than tomosynthesis, with a percentage of false positive recall rate matching the first study (interim report). However, this percentage is significantly higher with the addition of echography in the second study [10,51]. In our study, only one of the eight women with visible tumors detected using 3D prone ABUS had axillary metastases, indicating tumors at a later stage; we did not detect any invasive lobular cancers, only mucus neoplasm [29]. Our study highlights the benefits achieved concerning dense breast cancer detection rates when combining mammography and 3D ABUS. Previous studies have already shown those benefits and improvements, but our study differed from these since, for the first time. 3D prone ABUS technology was used. However, we do suggest that there are some limitations to our study. It is a descriptive prospective mono-center study and, as such, it does not include a high number patients or an analysis of interval cancer cases; we also did not include patient mortality rates, but this was not a primary aim of this study. As previously stated, what makes a difference in the literature is that this is the first study in which 3D prone ABUS performing technology has been used and evaluated. Carrying out the exam with 3D prone ABUS did not involve particular problems related to patient discomfort.

## 5. Conclusions

To conclude, we can assert that, for women with dense breasts, the cancer screening process is enhanced by combining 3D prone ABUS and FFDSM. Through this combination, cancer detection rates increase, but they also do so within an admissible recall rate.

The above has been already stated in previous studies, and we need more studies and further investigations to better assess the combined methodologies’ advantages and benefits, as well as their feasibility in terms of costs in screening programs for women with dense breasts or very dense breasts. The results of the present contribution suggest that the total performances of the 3D prone ABUS system are substantially comparable to those of the 3D ABUS systems in which patients are supine. That said, to confirm this notion, further comparative studies with larger numbers of patients are needed.

## Figures and Tables

**Table 1:** Patient’s age and American College of Radiology (ACR) category.

	ACR Density Categories		Total Patients	*p* Value *
	ACR3 N.729 (%)	ACR4 N.436 (%)	N.1165 (%)	
AGE GROUP				0.145
40–50	415 (75%)	275 (61%)	690 (68%)	
50–60	207 (20%)	118 (29%)	325 (25%)	
60–75	107 (5%)	43 (10%)	150 (7%)	

* *p* Value derived from *t*-test and chi-square test for continuous and categorical variables.

**Table 2:** Mammography and 3D prone ABUS breast cancer detection.

Features	Mammography	3D Prone ABUS	Difference	*p* Value
Cancer detected	4	8	4	
ACR density				
ACR3	3 (25%)	4 (50%)	1 (75%)	
ACR4	1 (75%)	4 (50%)	3 (25%)	
Size of cancer (MM)				
Mean (SD)	16.6 (3.47)	16.96 (2.59)	0,99	*p* < 0.05
Median (Q₁, Q₃)	17.2 (16, 19.42)	17.85 (16.40, 19.65)	17.52	
Range (MIN, MAX)	12, 20	12.60, 20.04	12, 20.04	
Histological classification, N (%)				
Grade I	1 (75%)	4 (50%)	3 (25%)	
Grade II	2 (50%)	3 (30%)	1 (75%)	
Grade III	1 (75%)	1 (10%)		

**Table 3:** Mammography and 3D prone ABUS screening results.

Characteristics	Mammography		Mammography + 3D Prone ABUS		Difference		*p* Value
Recalled women	17		31		14		
Cancer detected	4	(1.09–10.24)	8	(3.45–15.76)	4	(1.09–10.24)	*p* ˂ 0.001
Sensitivity (%)	58.8	(30.9–78.3)	93.5	(79.2–98.2)	34.7	(16.3–61.2)	*p* ˂ 0.001
Sensitivity including interval cancer (%)	35.2	(17.3–58.7)	67	(50.0–81.4)	31.8	(11.7– 54.6)	*p* ˂ 0.001
Specificity (%)	94	(73.0–98.0)	87	(71.0–94.8)	7	(4.3–8.8)	*p* ˂ 0.001
Cancer detection rate (‰)	3.4	(1.7–5.8)	6.8	(5.0–8.1)	3.4	(1.6–6.1)	*p* ˂ 0.007
Recall rate (‰)	14.5	(9.0–19.8)	26.6	(16.2–30.0)	12.1	(4.0–39.9)	*p* ˂ 0.001
Biopsy rate (‰)	7	(4.1–8.2)	14	(5.0–28.0)	7	(4.3–8.8)	*p* ˂ 0.001
PPV₁ (%)	68	(41.3–82.2)	24.8	(13.7–43.2)	43.2	(21.3–67.5)	*p* ˂ 0.001
PPV₂ and PPV₃ (%) ^b^	58.8	(30.9–78.3)	41	(26.4–59.2)	17.8	(7.5–47.5)	*p* ˂ 0.001
NPV at screening (%)	99.9	(81.5–99.9)	100	(_–_)	0.1	(0.1–2.7)	*p* ˂ 0.9203
NPV including interval cancers (%)	99.5	(73.1–99.8)	99.3	(83.8–99.4)	0.2	(0.12–3.1)	*p* ˂ 0.8415

Note: numbers in parentheses are 95% confidence intervals. ^b^ PPV₂ and PPV₃ are identical due to the fact that all the recommended biopsies were performed.

**Table 4:** Distribution of cancers detected by 3D prone ABUS performed after negative mammography.

Case n.	Age	mm Cancer	Density	Morphology	pT	pN	pM	Histological Type
1	58	18.9	D3	Mass	t1c	0	0	Ductal NOS
2	41	21.8	D3	Mass	t2	1(mi) *	0	Ductal NOS
3	46	18.7	D4	Mass	t1c	0	0	Ductal NOS
4	52	19.6	D3	Mass	t1c	0	0	Ductal NOS
5	64	18.7	D4	Mass	t1c	0	0	Mucinous
6	55	10.0	D3	Mass	t1b	0	0	Ductal NOS
7	42	19.8	D4	Mass	t1c	0	0	Ductal NOS
8	48	18.6	D4	Mass	t1c	0	0	Ductal NOS

* micrometastasis.

**Table 5:** Radiologists’ (N. 3) BI-RADS assessment of variance.

		BI-RADS-1	BI-RADS-2	BI-RADS-3	BI-RADS-4	BI-RADS-5	Total
Radiologist 1		30	49	18	7	8	112
Radiologist 2		29	50	18	7	8	112
Radiologist 3		48	39	9	8	8	112
Mean		35.67	46.00	40.83	32.22	26.00	
K Fleiss value	0.720						
Mean observed value	0.802						
Random mean value	0.290						

## Data Availability

The data presented in this study are available on request from the corresponding author. The data are not publicly available because are propriety of Università della Campania “Luigi Vanvitelli”, Napoli, Italy.

## Abbreviations

3D ABUS: three dimensional automated breast ultrasound system; ACR: American college of radiology; BI-RADS: breast imaging reporting and data system; CI: confidence interval; FFDM: full field digital mammography; HHUS; manual hand held ultrasound(traditional system with operator); DCIS: ductal carcinoma in situ; MRI: magnetic resonance imaging; CC: craniocaudal screening in mammography; MLO: mediolateral oblique screening in mammography; ML: mediolateral screening in mammography; FFDSM: full field digital screening mammography; PPV: positive predictive value; NPV: negative predictive value; MACE: Major adverse cardiac events; CAD: computed aided detection.
